# Relationship between headaches and tinnitus in a Swedish study

**DOI:** 10.1038/s41598-020-65395-1

**Published:** 2020-05-22

**Authors:** Alessandra Lugo, Niklas K. Edvall, Andra Lazar, Golbarg Mehraei, Jose-Antonio Lopez-Escamez, Jan Bulla, Inger Uhlen, Barbara Canlon, Silvano Gallus, Christopher R. Cederroth

**Affiliations:** 1 Department of Environmental Health Sciences, Istituto di Ricerche Farmacologiche Mario Negri IRCCS, Milan, Italy; 2 Laboratory of Experimental Audiology, Department of Physiology and Pharmacology, Karolinska Institutet, 171 77 Stockholm, Sweden; 3 Hörsel och balansmottagningen, Karolinska Universitetssjukhuset, Stockholm, Sweden; 4 Decibel Therapeutics Inc., Boston, USA; 5 Otology & Neurotology Group, Department of Genomic Medicine, Pfizer - Universidad de Granada - Junta de Andalucía Centro de Genómica e Investigación Oncológica (GENYO), PTS, Avenida de la Ilustración 114, 18016 Granada, Spain; 6 Department of Otolaryngology, Hospital Universitario Virgen de las Nieves, Instituto de Investigacion Biosanitaria ibs.GRANADA, Granada, Spain; 7 University of Bergen, Bergen, Norway; 8 University of Regensburg, Regensburg, Germany

**Keywords:** Headache, Risk factors, Auditory system

## Abstract

The heterogeneity of tinnitus is likely accounting for the lack of effective treatment approaches. Headaches have been related to tinnitus, yet little is known on how headaches impact tinnitus. We use cross-sectional data from the Swedish Tinnitus Outreach Project to i) evaluate the association between headaches and tinnitus (n = 1,984 cases and 1,661 controls) and ii) investigate the phenotypic characteristics of tinnitus subjects with tinnitus (n = 660) or without (n = 1,879) headaches. In a multivariable logistic regression model, headache was significantly associated with any tinnitus (odds ratio, OR = 2.61) and more so with tinnitus as a big problem (as measured by the tinnitus functional index, TFI ≥ 48; OR = 5.63) or severe tinnitus (using the tinnitus handicap inventory, THI ≥ 58; OR = 4.99). When focusing on subjects with tinnitus, the prevalence of headaches was 26% and reached 40% in subjects with severe tinnitus. A large number of socioeconomic, phenotypic and psychological characteristics differed between headache and non-headache subjects with any tinnitus. With increasing tinnitus severity, fewer differences were found, the major ones being vertigo, neck pain and other pain syndromes, as well as stress and anxiety. Our study suggests that headaches could contribute to tinnitus distress and potentially its severity.

## Introduction

Subjective tinnitus is the perception of sounds in absence of external stimuli. Its prevalence ranges from 5–43%, and for about one in ten with tinnitus, the impact is such that individuals seek medical help^1^. This so-called clinically significant tinnitus is strongly associated with anxiety, depression and stress^2–6^. Recent studies revealed an increased risk in suicide attempts in subjects with tinnitus^7,8^. The combination of these psychological comorbidities with tinnitus has a non-negligible impact on quality of life^3^. Available treatments are of limited efficacy^9–11^, subsequently leading to substantial healthcare costs^12^. It is now agreed that the heterogeneity of tinnitus could be grounds to treatment failures^13^, which has led to research programs aimed at identifying means of classifying tinnitus subtypes^14^. For instance, hyperacusis^15^ and temporomandibular complaints^16,17^ have been proposed as potential criteria for subtyping tinnitus.

Headaches have also been recently suggested as an important co-factor for tinnitus subtyping^18^, but there is limited knowledge on how headaches impact tinnitus. Similar to tinnitus, headaches are very heterogeneous. Migraine is a common primary headache disorder that can be experienced with or without aura, which are defined by episodes of transient focal neurological symptoms. The neural mechanisms of migraine have been reviewed elsewhere^19^. Recent studies reveal a high prevalence of headache (26–56%) in children, adolescents and patients with tinnitus^18,20,21^. Indeed, cross-sectional studies show that headache is associated with tinnitus^22,23^, and this association is greater in presence of migraine headache with aura^24^. As headache are very prevalent in the population, the co-occurrence of tinnitus and headache or migraine could be coincidental. However, two longitudinal studies show that a history of migraine is a risk factor for the development of tinnitus^25,26^.

The pathophysiology of tinnitus is thought to derive from a failure to adapt to missing sensory information^27^. Concomitant with hearing loss, the brain attempts to compensate for the diminished sensory input causing an increased neural activity in the auditory pathway^28^. Other structures such as the amygdala have been shown to interact with the auditory pathway in tinnitus patients^29^. Frontostriatal circuits, similar to those involved in chronic pain, have also been evidenced in tinnitus^30^. Auditory steady-state responses captured using magnetoencephalography reveal that the synchrony between different brain regions, such as the right parietal area and the anterior cingulate cortex correlates strongly with tinnitus intrusiveness^31^. However, headache and tinnitus also share common mechanisms characterized by changes in oscillatory activity called thalamocortical dysrhythmia^32–34^. Furthermore, alterations in the trigeminal system form a basis for the development of headaches and migraines^35,36^ as well as for tinnitus^16,37^.

Using data from the Tinnitus Research Initiative (TRI) database, Langguth *et al*. evaluated the impact of self-reported headache in tinnitus patients and found that a larger proportion of those with headache complaints were female, with more frequent comorbid vertigo, temporomandibular joint complaints, neck pain and other pain disorders, and depressive symptoms^18^. Although a number of publications deal with the relationship between tinnitus and headache, only a limited number of these provide a measure of association between headaches and the risk of tinnitus after adjustment for potential confounding factors^24,38,39^. To our knowledge, none have addressed the risk with severe tinnitus. Here, we investigate this relationship in the Swedish Tinnitus Outreach Projet (STOP), a study designed to investigate the risk factors for tinnitus, and further analyze the phenotypic traits accompanying tinnitus when combined with headaches.

## Results

### Association study

We subjected STOP participants with (n = 1,984) and without tinnitus (n = 1,661) to the ESIT-SQ in order to evaluate the association between headaches and tinnitus. Within tinnitus cases, 13.3% reported having tinnitus as a big problem according to the TFI cut-off, and 7.9% had severe tinnitus according to the THI cut-off. Subjects with headaches more frequently reported any tinnitus (multivariate OR, 2.61; 95% CI, 2.19–3.12), tinnitus as a big problem (OR, 5.63; 95% CI, 4.10–7.72) and severe tinnitus (OR, 4.99; 95% CI, 3.41–7.32; Table 1). These associations persisted to a similar degree when considering the further adjustment for hearing ability (model 2, Table 1). We also evaluated the association between headache and tinnitus stratifying by sex. The relationship with any tinnitus persisted to the same degree in men (OR, 3.21; 95% CI, 2.22–4.64) and in women (OR, 2.49; 95% CI, 2.03–3.06). The association was greater in men, although not significantly different from women, both for tinnitus as a big problem (men: OR, 7.28; 95% CI, 4.19–12.6 and women: OR, 5.51; 95% CI, 3.69–8.22) and for severe tinnitus (men: OR, 8.14; 95% CI, 4.22–15.7 and women: OR, 4.20; 95% CI, 2.59–6.80). Thus, with increasing severity, the relationship between headache and tinnitus becomes stronger.

**Table 1:** Odds ratios (OR) for any tinnitus, tinnitus as a big problem (TFI ≥ 48) and severe tinnitus (THI ≥ 58), and corresponding 95% confidence intervals (CI), according to headache.

	No tinnitus	Any tinnitus			Tinnitus as a big problem (TFI ≥ 48)			Severe tinnitus (THI ≥ 58)		
	N (%)	N (%)	OR* (95% CI)	OR° (95% CI)	N (%)	OR* (95% CI)	OR° (95% CI)	N (%)	OR* (95% CI)	OR° (95% CI)
***Both sexes***										
Total	1661 (100.0)	1984 (100.0)	—	—	263 (100.0)	—	—	157 (100.0)	—	—
**Headache**										
No	1421 (85.6)	1458 (73.5)	1^	1^	156 (59.3)	1^	1^	94 (59.9)	1^	1^
Yes	240 (14.4)	526 (26.5)	**2.61 (2.19–3.12)**	**2.19 (1.81–2.64)**	107 (40.7)	**5.63 (4.10–7.72)**	**4.29 (2.96–6.22)**	63 (40.1)	**4.99 (3.41–7.32)**	**3.80 (2.43–5.94)**
***Men***										
Total	563 (100.0)	947 (100.0)	—	—	127 (100.0)	—	—	71 (100.0)	—	—
**Headache**										
No	524 (93.1)	768 (81.1)	1^	1^	87 (68.5)	1^	1^	46 (64.8)	1^	1^
Yes	39 (6.9)	179 (18.9)	**3.21 (2.22–4.64)**	**2.64 (1.80–3.87)**	40 (31.5)	**7.28 (4.19–12.63)**	**6.09 (3.26–11.35)**	25 (35.2)	**8.14 (4.22–15.7)**	**7.47 (3.55–15.7)**
***Women***										
Total	1098 (100.0)	1034 (100.0)	—	—	135 (100.0)	-	-—	85 (100.0)	—	—
**Headache**										
No	897 (81.7)	689 (66.6)	1^	1^	68 (50.4)	1^	1^	47 (55.3)	1^	1^
Yes	201 (18.3)	345 (33.4)	**2.49 (2.03–3.06)**	**2.09 (1.68–2.61)**	67 (49.6)	**5.51 (3.69–8.22)**	**4.15 (2.56–6.73)**	38 (44.7)	**4.20 (2.59–6.80)**	**2.98 (1.66–5.34)**

TFI: Tinnitus Functional Index; THI: Tinnitus Handicap Inventory.

*ORs were estimated using unconditional multiple logistic regression models after adjustment for sex, age, and level of education (model 1). Estimates in bold are statistically significant at 0.05 level.

°ORs were estimated using unconditional multiple logistic regression models after adjustment for sex, age, level of education, and hearing ability (model 2). Estimates in bold are statistically significant at 0.05 level.

^Reference category.

### Tinnitus phenotyping study

We next focused on tinnitus subjects only. Among 2,539 subjects with any tinnitus, 26% reported headache. This proportion increased for individuals reporting tinnitus as a big problem (36%), and for those with severe tinnitus (40%). Table 2 describes the sociodemographic characteristics of these three groups. There was a greater proportion of women in the any tinnitus groups with headaches in comparison to those without headaches. This gender bias survived the adjustment for multiple comparisons in the “any tinnitus” group but not in groups with more severe tinnitus. Any tinnitus subjects with headaches significantly differed from those without headaches in age, income, and employment status (*p* = <0.0001) but not in marital status or education level. In contrast, in the samples with severe tinnitus, no differences were found in all sociodemographic variables after correction for multiple comparisons. We next used a set of questionnaires to assess the global impact of headache on tinnitus-related burden^6,40^. Subjects with any tinnitus who reported headache were found to have worse scores for all questionnaires (*p* < 0.0001, Table 3), with the exception of the numerical rating scale for awareness. In subjects with severe tinnitus, the only differences were found in the TFI, stress, anxiety measures, and physical and psychological quality of life (*p* = 0.039 to 0.025 after correction for multiple comparisons). Thus, headaches contribute to greater psychological burden in tinnitus subjects, regardless of the degree of severity.

**Table 2:** Sociodemographic and psychological characteristics for subjects with tinnitus with or without headache.

	Any Tinnitus		Tinnitus as a big problem		Severe tinnitus	
	Headache (n = 660) No (%)	No headache (n = 1879) No (%)	Headache (n = 125) No (%)	No Headache (n = 218) n (%)	Headache (n = 79) No (%)	No Headache (n = 119) n (%)
**Sex**	**χ²(1) = 94.59, p** < **0.0001 (<0.0001)**		**χ²(1) = 23.31, p** < **0.0001 (p** = **0.0003)**		χ²(1) = 6.09, p = 0.014 (0.082)	
Male	225 (34.1)	1054 (56.1)	43 (34.4)	134 (61.5)	31 (39.2)	68 (57.1)
Female	435 (65.9)	825 (43.9)	82 (65.6)	84 (38.5)	48 (60.8)	51 (42.9)
**Age Group**	**χ²(7) = 38.47, p** < **0.0001 (<0.0001)**		χ²(7) = 5.81, p = 0.5619 (0.5619)		χ²(7) = 2.84, p = 0.899 (0.899)	
<24	19 (2.9)	40 (2.1)	6 (4.8)	4 (1.8)	4 (5.1)	6 (5.1)
25–34	140 (21.3)	350 (18.7)	19 (15.3)	34 (15.7)	13 (16.7)	28 (23.7)
35–44	168 (25.6)	407 (21.7)	25 (20.2)	42 (19.4)	14 (18)	21 (17.8)
45–54	192 (29.2)	487 (26)	39 (31.5)	62 (28.6)	22 (28.2)	26 (22)
55–64	88 (13.4)	272 (14.5)	20 (16.1)	37 (17.1)	14 (18)	21 (17.8)
65–74	43 (6.5)	268 (14.3)	12 (9.7)	24 (11.1)	9 (11.5)	11 (9.3)
75–84	6 (0.9)	48 (2.6)	2 (1.6)	12 (5.5)	2 (2.6)	4 (3.4)
>85	1 (0.2)	3 (0.2)	1 (0.8)	2 (0.9)	0 (0)	1 (0.9)
**Marital Status**	χ²(4) = 7.84, p = 0.0974 (0.0974)		χ²(4) = 4.23, p = 0.3757 (0.45084)		χ²(4) = 4.36, p = 0.359 (0.539)	
Married	268 (40.6)	830 (44.2)	40 (32)	91 (41.7)	29 (36.7)	43 (36.1)
Living with partner	211 (32)	551 (29.3)	41 (32.8)	68 (31.2)	25 (31.7)	44 (37)
Single	132 (20)	320 (17)	28 (22.4)	41 (18.8)	16 (20.3)	25 (21)
Widow/Widower	5 (0.8)	26 (1.4)	3 (2.4)	3 (1.4)	1 (1.3)	3 (2.5)
Divorced	44 (6.7)	152 (8.1)	13 (10.4)	15 (6.9)	8 (10.1)	4 (3.4)
**Gross income**	**χ²(3) = 37.56, p** < **0.0001 (<0.0001)**		**χ²(3) = 12.72, p** = **0.0053 (0.0159)**		χ²(3) = 5.08, p = 0.166 (0.332)	
0–200 000 SEK	90 (13.6)	215 (11.4)	27 (21.6)	36 (16.5)	17 (21.5)	25 (21)
200 001–450 000 SEK	368 (55.8)	877 (46.7)	72 (57.6)	110 (50.5)	49 (62)	60 (50.4)
450 001 SEK or more	162 (24.6)	706 (37.6)	14 (11.2)	59 (27.1)	7 (8.9)	24 (20.2)
Don’t know/don’t want to disclose	40 (6.1)	81 (4.3)	12 (9.6)	13 (6)	6 (7.6)	10 (8.4)
**Education Level**	χ²(3) = 6.8, p = 0.0785 (0.0942)		χ²(3) = 5.1, p = 0.1646 (0.2469)		χ²(3) = 8, p = 0.046 (0.138)	
Middle School	20 (3)	48 (2.6)	8 (6.4)	13 (6)	8 (10.1)	5 (4.2)
High School	138 (20.9)	397 (21.1)	34 (27.2)	70 (32.1)	23 (29.1)	37 (31.1)
University	424 (64.2)	1273 (67.8)	57 (45.6)	109 (50)	32 (40.5)	65 (54.6)
Other	78 (11.8)	161 (8.6)	26 (20.8)	26 (11.9)	16 (20.3)	12 (10.1)
**Employment**	**χ²(10) = 75.25, p** < **0.0001 (<0.0001)**		**χ²(8) = 19.2, p** = **0.0138 (0.0276)**		χ²(7) = 4.54, p = 0.716 (0.86)	
Don’t know	2 (0.3)	0 (0)	0 (0)	0 (0)	0 (0)	0 (0)
Employed	430 (65.2)	1140 (60.7)	59 (47.2)	111 (50.9)	39 (49.4)	59 (49.6)
Unemployed	14 (2.1)	17 (0.9)	7 (5.6)	2 (0.9)	4 (5.1)	2 (1.7)
Running my own business/Working as a partner in a company	67 (10.2)	249 (13.3)	8 (6.4)	30 (13.8)	4 (5.1)	14 (11.8)
Retired	44 (6.7)	286 (15.2)	15 (12)	37 (17)	11 (13.9)	14 (11.8)
Sick leave (for more than two month) or disability pension due to illness or disability	47 (7.1)	49 (2.6)	18 (14.4)	23 (10.6)	12 (15.2)	16 (13.5)
Parental leave (since two months or longer)	13 (2)	27 (1.4)	3 (2.4)	3 (1.4)	1 (1.3)	2 (1.7)
Student	28 (4.2)	79 (4.2)	10 (8)	10 (4.6)	7 (8.9)	10 (8.4)
Sabbatical	3 (0.5)	2 (0.1)	1 (0.8)	0 (0)	0 (0)	0 (0)
Housewife/-Husband	0 (0)	1 (0.1)	0 (0)	0 (0)	0 (0)	0 (0)
Other	12 (1.8)	29 (1.5)	4 (3.2)	2 (0.9)	1 (1.3)	2 (1.7)

Income refers to yearly income in SEK. Pairwise comparisons using Pearson’s Chi-square test are reported. Percentages (%) displayed refer to column percentages. P values adjusted for multiple comparisons are shown between parenthesis. Estimates in bold are statistically significant at 0.05 level.

**Table 3:** Questionnaire scores from subjects with tinnitus with or without headache.

	Any Tinnitus		Tinnitus as a big problem		Severe tinnitus	
	Headache (n = 660) mean (SD)	No headache (n = 1879) mean (SD)	Headache (n = 125) mean (SD)	No headache (n = 218) mean (SD)	Headache (n = 79) mean (SD)	No headache (n = 119) mean (SD)
NRS Lo	44 (25.5)	39.5 (25.3)	72.4 (19.3)	70.6 (19.1)	76.1 (18.4)	72.8 (20.0)
	**3.89, p** = **0.0001 (0.0001, −0.10**^†^**)**		0.87, p = 0.3829 (0.4248, −0.06^†^)		1.08, p = 0.2816 (0.352, −0.09^†^)	
NRS Aw	35.3 (32.3)	32.9 (31.1)	73.5 (27.5)	74 (24.6)	76.1 (25.5)	75 (25.4)
	1.87, p = 0.0612 (0.0612, −0.05^†^)		0.36, p = 0.722 (0.722, −0.02^†^)		1.08, p = 0.2816 (0.352, −0.03^†^)	
NRS An	23.3 (27.4)	19 (24.8)	62.4 (28.0)	58.9 (26.2)	68.7 (27.2)	64.4 (26.9)
	**4.6, p** < **0.0001 (<0.0001**, −**0.12**^†^**)**		1.26, p = 0.2059 (0.2574, −0.08^†^)		1.29, p = 0.1978 (0.2697, −0.11^†^)	
THI	26.6 (22.9)	19.9 (19.2)	62.4 (19.0)	57.4 (18.3)	75.3 (11.4)	72 (12)
	**7.09, p** < **0.0001 (<0.0001**, −**0.19**^†^**)**		**2.3, p** = **0.0212 (0.0353**, −**0.15**^†^**)**		2.15, p = 0.0319 (0.0684, −0.18^†^)	
TFI	26.6 (23.2)	20.2 (19.8)	65.8 (12.4)	62.1 (11.9)	69.6 (14.2)	64.4 (16.2)
	**6.07, p** < **0.0001 (<0.0001, -0.16**^†^**)**		**2.91, p** = **0.0036 (0.0068, -0.18**^†^**)**		**2.48, p** = **0.0131 (0.0393, -0.21**^†^**)**	
PSQ	0.4 (0.2)	0.3 (0.2)	0.5 (0.2)	0.5 (0.2)	0.6 (0.2)	0.5 (0.2)
	**13.08, p** < **0.0001 (<0.0001**, −**0.34**^†^**)**		**4.54, p** < **0.0001 (0.0003**, −**0.29**^†^**)**		**2.94, p** = **0.0033 (0.0248**, −**0.25**^†^**)**	
HADS_A	7.8 (4.4)	5.5 (3.9)	10.6 (4.4)	8.7 (4.5)	11.8 (4.2)	10.1 (4.5)
	**11.59, p** < **0.0001 (<0.0001**, −**0.3**^†^**)**		**3.58, p** = **0.0003 (0.0011**, −**0.23**^†^**)**		**2.58, p** = **0.01 (0.0393**, −**0.22**^†^**)**	
HADS_D	4.7 (3.7)	3.3 (3.2)	7.6 (3.9)	6.4 (3.9)	8.3 (4.0)	7.5 (4.1)
	**9.15, p** < **0.0001 (<0.0001**, −**0.24**^†^**)**		**2.99, p** = **0.0028 (0.006**, −**0.19**^†^**)**		1.66, p = 0.0962 (0.1566, −0.14^†^)	
FTQ	5.1 (2.9)	4.6 (2.6)	8.1 (3.2)	8.3 (2.9)	9 (3.0)	9.6 (2.7)
	**3.85, p** = **0.0001 (0.0001**, −**0.1**^†^**)**		−0.85, p = 0.3965 (0.4248, 0.05^†^)		−1.63, p = 0.1033 (0.1566, 0.14^†^)	
TCS	14.9 (11.5)	11.7 (9.8)	28.1 (11.0)	26.9 (9.4)	31.4 (9.6)	30.4 (8.7)
	**6.02, p** < **0.0001 (<0.0001**, −**0.16**^†^**)**		1.53, p = 0.1259 (0.1717, −0.1^†^)		0.93, p = 0.3542 (0.4087, −0.08^†^)	
HQ	20.2 (9.3)	15.5 (8.9)	26.3 (7.7)	23.3 (8.8)	27.3 (8.0)	25.5 (8.8)
	**11, p** < **0.0001 (<0.0001**, −**0.29**^†^**)**		**3.14, p** = **0.0017 (0.0043**, −**0.2**^†^**)**		1.62, p = 0.1044 (0.1566, −0.14^†^)	
QoL Phy	14.4 (2.9)	16.1 (2.5)	12 (2.7)	13.7 (2.7)	11.6 (2.8)	13 (2.7)
	−**13.63, p** < **0.0001 (<0.0001, 0.36**^†^**)**		−**5.29, p** < **0.0001 (0.0003, 0.34**^†^**)**		−**3.14, p** = **0.0017 (0.0248, 0.26**^†^**)**	
QoL Psy	13.9 (2.9)	15.2 (2.5)	11.8 (2.9)	13 (2.8)	11.1 (2.8)	12.3 (2.8)
	−**10.32, p** < **0.0001 (<0.0001, 0.27**^†^**)**		−**3.55, p** = **0.0004 (0.0012, 0.23**^†^**)**		−**2.49, p** = **0.0129 (0.0393, 0.21**^†^**)**	
QoL Social	13.6 (3.2)	14.4 (3)	12.2 (3.6)	13.2 (3.3)	12.3 (3.2)	12.7 (3.6)
	−**5.26, p** < **0.0001 (<0.0001, 0.14**^†^**)**		−**2.25, p** = **0.0248 (0.0372, 0.14**^†^**)**		−0.58, p = 0.5591 (0.599, 0.05^†^)	
QoL Env	15.5 (2.4)	16.5 (2.1)	13.9 (2.4)	15 (2.6)	13.9 (2.5)	14.7 (2.7)
	−**9.81, p** < **0.0001 (<0.0001, 0.25**^†^**)**		−**3.99, p** < **0.0001 (0.0003, 0.26**^†^**)**		−2.25, p = 0.0245 (0.0613, 0.19^†^)	

Values are mean (± SD). Pairwise comparisons using Wilcoxon’s tests are reported below the compared values. P values adjusted for multiple comparisons are shown between parenthesis together with Cliff’s δ (noted †). Abbreviations: Numerical Ratings Score (NRS), Tinnitus loudness (Lo), Awareness (Aw), Annoyance (An), Tinnitus Handicap Inventory (THI), Tinnitus Functional Index (TFI), Fear of Tinnitus Questionnaire (FTQ), Tinnitus Catastrophising Scale (TCS), Hyperacusis Questionnaire (HQ), Perceived Stress Questionnaire (PSQ), Hospital Anxiety Depression Scales for Anxiety (HADS A) and depression (HADS D), Quality of Life (QoL) subscales from the World Health Organization: Physical (Phy), Psychological

(Psych), Social (Soc), and Environmental (Env). Estimates in bold are statistically significant at 0.05 level.

When evaluating the tinnitus phenotypic characteristics for the group with any tinnitus, differences were observed in onset-related events (*p* = 0.006), with subjects more frequently reporting stress as the cause of their tinnitus (Table 4). Such effects were not seen in subjects with tinnitus as a big problem or severe tinnitus. Differences were also found in tinnitus loudness variation from day to day and sound of tinnitus (*p* = 0.001 to 0.003). Pulsatility was also different between headache and non-headache subjects with “any tinnitus” (*p* < 0.0001), although this could be due to a larger proportion in the headache group not being able to define it as pulsatile or not. Sounds also had a greater impact on subjects with any tinnitus and headaches, with greater worsening when exposed to loud noise, greater intolerance to sounds, and sounds causing pain and discomfort (*p* = 0.002 to <0.0001, Tables 4 and 5). The impact of stress, bad night sleep and naps also differed between any tinnitus subjects with or without headaches. A greater proportion of somatosensory impact such as tinnitus being affected by head movement and touch, self-reporting of temporomandibular complaints, vertigo and dizziness, neck pain and other pain syndromes was also found in any tinnitus subjects with headache (*p* < 0.0001). More subjects with headaches reported a diagnosed disease and were under psychiatric treatment (*p* < 0.0001), and a greater occurrence of tinnitus in the family was also reported in the headache group (*p* = 0.03). The proportion of subjects with self-reported hearing problems, hearing aids, and the location of tinnitus (laterality), did not differ between the headache and non-headache groups suggesting a minimal contribution of hearing loss into migraine in tinnitus subjects. Interestingly, in the severe tinnitus group, only vertigo/dizziness, neck pain and other pain syndromes remained different between the headache and non-headache groups (*p* = 0.014 to 0.002, Table 5).

**Table 4:** Phenotypic characteristics for subjects with tinnitus with or without headache.

	Any Tinnitus		Tinnitus as a big problem		Severe tinnitus	
	Headache (n = 660) No (%)	No Headache (n = 1879) No (%)	Headache (n = 125) No (%)	No Headache (n = 218) No (%)	Headache (n = 79) No (%)	No Headache (n = 119) No (%)
**Tinnitus onset**	χ²(5) = 5.02, p = 0.414 (0.497)		χ²(5) = 1.31, p = 0.934 (0.946)		χ²(5) = 3.91, p = 0.562 (0.919)	
Don’t know	71 (10.8)	161 (8.6)	3 (2.4)	5 (2.3)	2 (2.5)	2 (1.7)
0 to 6 months	13 (2.0)	39 (2.1)	5 (4.0)	8 (3.7)	4 (5.1)	5 (4.2)
6 months to 3 years	105 (15.9)	265 (14.1)	17 (13.6)	37 (17.0)	14 (17.7)	24 (20.2)
3 to 10 years	183 (27.7)	546 (29.1)	32 (25.6)	61 (28.0)	21 (26.6)	31 (26.1)
10 to 20 years	180 (27.3)	559 (29.8)	39 (31.2)	64 (29.4)	18 (22.8)	38 (31.9)
More than 20 years	108 (16.4)	309 (16.4)	29 (23.2)	43 (19.7)	20 (25.3)	19 (16.0)
**Onset-related events**	χ²(6) = 19.68, p = 0.003 (0.006)		χ²(6) = 8.52, p = 0.203 (0.405)		χ²(6) = 2.26, p = 0.894 (0.919)	
Loud blast of sound	224 (33.9)	759 (40.4)	35 (28)	71 (32.6)	23 (29.1)	33 (27.7)
Stress	117 (17.7)	244 (13)	21 (16.8)	35 (16.1)	12 (15.2)	23 (19.3)
Change in hearing	41 (6.2)	93 (5.0)	16 (12.8)	19 (8.7)	10 (12.7)	10 (8.4)
Head trauma	6 (0.9)	15 (0.8)	0 (0.0)	2 (0.9)	0 (0.0)	1 (0.8)
Whiplash	10 (1.5)	11 (0.6)	4 (3.2)	1 (0.5)	1 (1.3)	1 (0.8)
Other	90 (13.6)	261 (13.9)	20 (16)	46 (21.1)	17 (21.5)	24 (20.2)
Don’t know	172 (26.1)	496 (26.4)	29 (23.2)	44 (20.2)	16 (20.3)	27 (22.7)
**Tinnitus occurrence**	χ²(1) = 3.15, p = 0.076 (0.104)		χ²(1) = 4.98, p = 0.026 (0.086)		χ²(1) = 0.01, p = 0.913 (0.919)	
Occasionally (now and then)	308 (46.7)	802 (42.7)	17 (13.6)	14 (6.4)	5 (6.3)	8 (6.7)
Always (all the time)	352 (53.3)	1077 (57.3)	108 (86.4)	204 (93.6)	74 (93.7)	111 (93.3)
**Time of the day of tinnitus emergence**	**χ²(6) = 13.8, p** = **0.032 (0.05)**		χ²(6) = 4.71, p = 0.581 (0.758)		χ²(6) = 3.05, p = 0.802 (0.919)	
Don’t know	350 (53)	1012 (53.9)	51 (40.8)	85 (39)	28 (35.4)	35 (29.4)
When awakening	34 (5.2)	123 (6.6)	14 (11.2)	31 (14.2)	13 (16.5)	22 (18.5)
In the morning	12 (1.8)	35 (1.9)	6 (4.8)	5 (2.3)	3 (3.8)	4 (3.4)
Around noon	37 (5.6)	125 (6.7)	12 (9.6)	24 (11.0)	9 (11.4)	16 (13.5)
In the afternoon	31 (4.7)	93 (5.0)	7 (5.6)	19 (8.7)	6 (7.6)	16 (13.5)
In the evening	71 (10.8)	238 (12.7)	21 (16.8)	38 (17.4)	15 (19.0)	17 (14.3)
Before sleeping	125 (18.9)	253 (13.5)	14 (11.2)	16 (7.3)	5 (6.3)	9 (7.6)
**Perceiving the onset of tinnitus**	χ²(2) = 6.12, p = 0.047 (0.067)		χ²(2) = 0.59, p = 0.744 (0.88)		χ²(2) = 0.23, p = 0.891 (0.919)	
Don’t know	164 (24.9)	494 (26.3)	14 (11.2)	22 (10.1)	6 (7.6)	7 (5.9)
Gradual	313 (47.4)	790 (42.0)	55 (44.0)	89 (40.8)	29 (36.7)	45 (37.8)
Abrupt	183 (27.7)	595 (31.7)	56 (44.8)	107 (49.1)	44 (55.7)	67 (56.3)
**Pulsatility**	**χ²(3) = 22.44, p** < **0.0001 (0.0001)**		**χ²(3) = 12.08, p** = **0.007 (0.03)**		χ²(3) = 5.21, p = 0.157 (0.485)	
Don’t know	77 (11.7)	138 (7.3)	20 (16.0)	22 (10.1)	5 (6.3)	14 (11.8)
Yes, with heart beat	56 (8.5)	124 (6.6)	19 (15.2)	17 (7.8)	14 (17.7)	10 (8.4)
Yes, different from heart beat	35 (5.3)	62 (3.3)	15 (12.0)	16 (7.3)	10 (12.7)	13 (10.9)
No	492 (74.6)	1555 (82.8)	71 (56.8)	163 (74.8)	50 (63.3)	82 (68.9)
**Location of tinnitus**	χ²(6) = 13.58, p = 0.035 (0.052)		χ²(6) = 4.84, p = 0.565 (0.758)		χ²(6) = 4.97, p = 0.548 (0.919)	
Right ear	50 (7.6)	147 (7.8)	10 (8.0)	22 (10.1)	6 (7.6)	11 (9.2)
Left ear	45 (6.8)	179 (9.5)	14 (11.2)	15 (6.9)	13 (16.5)	10 (8.4)
Both ears, worse in right	141 (21.4)	305 (16.2)	24 (19.2)	35 (16.1)	13 (16.5)	24 (20.2)
Both ears, worse in left	110 (16.7)	316 (16.8)	21 (16.8)	49 (22.5)	15 (19)	27 (22.7)
Both ears equally	197 (29.9)	622 (33.1)	32 (25.6)	51 (23.4)	15 (19)	20 (16.8)
Inside the head	108 (16.4)	289 (15.4)	19 (15.2)	40 (18.4)	12 (15.2)	23 (19.3)
Elsewhere	9 (1.4)	21 (1.1)	5 (4.0)	6 (2.8)	5 (6.3)	4 (3.4)
**Sound of tinnitus**	**χ²(9) = 27.04, p** = **0.001 (0.003)**		**χ²(7) = 22.48, p** = **0.002 (0.013)**		χ²(7) = 14.21, p = 0.048 (0.204)	
Tone	123 (18.9)	414 (22.2)	13 (10.7)	21 (9.7)	10 (13)	12 (10.3)
Noise	65 (10.0)	217 (11.6)	9 (7.4)	26 (12.0)	7 (9.1)	9 (7.7)
Crickets	18 (2.8)	71 (3.8)	2 (1.6)	7 (3.2)	2 (2.6)	5 (4.3)
Heartbeat	5 (0.8)	6 (0.3)	1 (0.8)	1 (0.5)	2 (2.6)	0 (0.0)
Beeping	67 (10.3)	233 (12.5)	6 (4.9)	34 (15.7)	4 (5.2)	17 (14.5)
Morse Code	0 (0.0)	4 (0.2)	0 (0.0)	0 (0.0)	0 (0.0)	0 (0.0)
An alarm	9 (1.4)	11 (0.6)	4 (3.3)	2 (0.9)	3 (3.9)	2 (1.7)
Other	11 (1.7)	62 (3.3)	1 (0.8)	13 (6)	1 (1.3)	11 (9.4)
Don’t know	1 (0.2)	1 (0.1)	0 (0)	0 (0.0)	0 (0.0)	0 (0.0)
Complex	352 (54.1)	848 (45.4)	86 (70.5)	112 (51.9)	48 (62.3)	61 (52.1)
**Tinnitus loudness variation from day to day**	**χ²(5) = 23.14, p** = **0 (0.001)**		χ²(5) = 3.53, p = 0.62 (0.775)		χ²(5) = 4.41, p = 0.493 (0.919)	
Don’t know	36 (5.5)	127 (6.8)	4 (3.2)	6 (2.8)	1 (1.3)	2 (1.7)
Never	38 (5.8)	154 (8.2)	10 (8.0)	18 (8.3)	6 (7.6)	6 (5.0)
Seldom	79 (12.0)	301 (16.0)	19 (15.2)	35 (16.1)	11 (13.9)	14 (11.8)
Sometimes	241 (36.5)	710 (37.8)	45 (36.0)	94 (43.1)	27 (34.2)	54 (45.4)
Often	186 (28.2)	397 (21.1)	30 (24.0)	47 (21.6)	19 (24.1)	30 (25.2)
Always	80 (12.1)	190 (10.1)	17 (13.6)	18 (8.3)	15 (19)	13 (10.9)
**Pitch of tinnitus**	χ²(4) = 0.12, p = 0.998 (0.998)		χ²(4) = 1.74, p = 0.783 (0.88)		χ²(4) = 2.55, p = 0.636 (0.919)	
Don’t know	27 (4.1)	72 (3.8)	4 (3.2)	5 (2.3)	2 (2.5)	3 (2.5)
Very high frequency	157 (23.8)	450 (24.0)	39 (31.2)	64 (29.4)	30 (38.0)	38 (31.9)
High frequency	293 (44.4)	838 (44.6)	54 (43.2)	101 (46.3)	30 (38.0)	47 (39.5)
Medium frequency	128 (19.4)	366 (19.5)	24 (19.2)	36 (16.5)	14 (17.7)	20 (16.8)
Low frequency	55 (8.3)	153 (8.1)	4 (3.2)	12 (5.5)	3 (3.8)	11 (9.2)
**Reduction of tinnitus by music or environmental sounds**	χ²(2) = 0.48, p = 0.787 (0.827)		χ²(2) = 2.02, p = 0.364 (0.682)		χ²(2) = 6.16, p = 0.046 (0.204)	
Don’t know	171 (25.9)	470 (25.0)	23 (18.4)	29 (13.3)	15 (19)	10 (8.4)
Yes	367 (55.6)	1074 (57.2)	68 (54.4)	133 (61.0)	45 (57.0)	67 (56.3)
No	122 (18.5)	335 (17.8)	34 (27.2)	56 (25.7)	19 (24.1)	42 (35.3)
**Worsening of tinnitus by loud noise**	**χ²(2) = 14.57, p** = **0.001 (0.002)**		χ²(2) = 1.36, p = 0.506 (0.758)		χ²(2) = 0.7, p = 0.703 (0.919)	
Don’t know	181 (27.4)	525 (27.9)	30 (24.0)	47 (21.6)	21 (26.6)	27 (22.7)
Yes	357 (54.1)	881 (46.9)	77 (61.6)	129 (59.2)	47 (59.5)	71 (59.7)
No	122 (18.5)	473 (25.2)	18 (14.4)	42 (19.3)	11 (13.9)	21 (17.7)
**Tinnitus affected by head movement or touch**	**χ²(2) = 38.19, p** < **0.0001 (0.0001)**		χ²(2) = 6.91, p = 0.032 (0.095)		χ²(2) = 0.8, p = 0.67 (0.919)	
Don’t know	157 (23.8)	361 (19.2)	15 (12.0)	35 (16.1)	8 (10.1)	12 (10.1)
Yes	210 (31.8)	426 (22.7)	61 (48.8)	75 (34.4)	42 (53.2)	56 (47.1)
No	293 (44.4)	1092 (58.1)	49 (39.2)	108 (49.5)	29 (36.7)	51 (42.9)
**Tinnitus affected by nap**	**χ²(3) = 9.61, p** = **0.022 (0.037)**		χ²(3) = 1.99, p = 0.574 (0.758)		χ²(3) = 0.5, p = 0.919 (0.919)	
Don’t know	373 (56.5)	1068 (56.8)	43 (34.4)	88 (40.4)	25 (31.7)	42 (35.3)
It mainly worsens my tinnitus	15 (2.3)	38 (2.0)	7 (5.6)	16 (7.3)	8 (10.1)	12 (10.1)
It mainly reduces my tinnitus	199 (30.2)	633 (33.7)	62 (49.6)	93 (42.7)	39 (49.4)	53 (44.5)
It has no effect	73 (11.1)	140 (7.5)	13 (10.4)	21 (9.6)	7 (8.9)	12 (10.1)
**Tinnitus affected by bad nights sleep**	**χ²(5) = 51.73, p** < **0.0001 (0.0001)**		χ²(5) = 7.62, p = 0.179 (0.383)		χ²(5) = 6.29, p = 0.279 (0.76)	
Don’t know	257 (38.9)	704 (37.5)	25 (20)	42 (19.3)	14 (17.7)	15 (12.6)
Never	53 (8)	315 (16.8)	6 (4.8)	24 (11.0)	3 (3.8)	10 (8.4)
Seldom	43 (6.5)	154 (8.2)	9 (7.2)	20 (9.2)	6 (7.6)	12 (10.1)
Sometimes	145 (22)	419 (22.3)	28 (22.4)	58 (26.6)	14 (17.7)	33 (27.7)
Often	122 (18.5)	221 (11.8)	39 (31.2)	54 (24.8)	27 (34.2)	33 (27.7)
Always	40 (6.1)	66 (3.5)	18 (14.4)	20 (9.2)	15 (19)	16 (13.5)
**Tinnitus affected by stress**	**χ²(3) = 69.4, p** < **0.0001 (0.0001)**		χ²(2) = 7.5, p = 0.024 (0.086)		χ²(2) = 1.43, p = 0.491 (0.919)	
Don’t know	211 (32)	677 (36)	23 (18.4)	50 (22.9)	12 (15.2)	20 (16.8)
Yes, it worsens my tinnitus	369 (55.9)	738 (39.3)	90 (72)	127 (58.3)	62 (78.5)	86 (72.3)
Yes, it reduces my tinnitus	79 (12)	460 (24.5)	12 (9.6)	41 (18.8)	5 (6.3)	13 (10.9)
No, it has no effect	1 (0.2)	4 (0.2)	0 (0.0)	0 (0.0)	0 (0.0)	0 (0.0)
**Tinnitus affected by medication**	χ²(2) = 0.95, p = 0.623 (0.692)		χ²(2) = 0.11, p = 0.946 (0.946)		χ²(2) = 0.32, p = 0.854 (0.919)	
Don’t know	482 (73)	1380 (73.4)	83 (66.4)	141 (64.7)	46 (58.2)	74 (62.2)
Yes	20 (3.0)	44 (2.3)	9 (7.2)	16 (7.3)	9 (11.4)	12 (10.1)
No	158 (23.9)	455 (24.2)	33 (26.4)	61 (28.0)	24 (30.4)	33 (27.7)
**Contacted a clinician due to tinnitus**	χ²(2) = 1.14, p = 0.566 (0.653)		χ²(2) = 1.88, p = 0.391 (0.69)		χ²(2) = 0.18, p = 0.916 (0.919)	
No	415 (62.9)	1209 (64.3)	32 (25.6)	43 (19.7)	11 (13.9)	17 (14.3)
Yes, because of curiosity	32 (4.9)	102 (5.4)	3 (2.4)	8 (3.7)	2 (2.5)	2 (1.7)
Yes, because I sought for help	213 (32.3)	568 (30.2)	90 (72.0)	167 (76.6)	66 (83.5)	100 (84.0)
**Number of tinnitus treatments**	χ²(3) = 3.18, p = 0.365 (0.456)		χ²(3) = 0.83, p = 0.842 (0.902)		χ²(3) = 2.29, p = 0.514 (0.919)	
None	563 (85.3)	1651 (87.9)	80 (64)	134 (61.5)	42 (53.2)	61 (51.3)
1	41 (6.2)	96 (5.1)	17 (13.6)	30 (13.8)	14 (17.7)	17 (14.3)
2–4	42 (6.4)	94 (5.0)	19 (15.2)	32 (14.7)	16 (20.3)	22 (18.5)
5 or more	14 (2.1)	38 (2.0)	9 (7.2)	22 (10.1)	7 (8.9)	19 (16)
**Tinnitus occurence in family**	**χ²(1) = 5.64, p** = **0.018 (0.031)**		χ²(1) = 0.52, p = 0.471 (0.744)		χ²(1) = 0.05, p = 0.832 (0.919)	
No	518 (78.5)	1553 (82.7)	89 (71.2)	163 (74.8)	56 (70.9)	86 (72.3)
Yes	142 (21.5)	326 (17.4)	36 (28.8)	55 (25.2)	23 (29.1)	33 (27.7)

Pairwise comparisons using Pearson’s Chi-square test are reported. Percentages (%) displayed refer to column percentages. P values adjusted for multiple comparisons are shown between parenthesis. Estimates in bold are statistically significant at 0.05 level.

**Table 5:** Comorbidities in subjects with tinnitus with or without headache.

	Any Tinnitus		Tinnitus as a big problem		Severe tinnitus	
	Headache (n = 660) No (%)	No Headache (n = 1879) No (%)	Headache (n = 125) No (%)	No Headache (n = 218) No (%)	Headache (n = 79) No (%)	No Headache (n = 119) No (%)
**Hearing problem**	χ²(2) = 2.86, p = 0.24 (0.313)		χ²(2) = 1.57, p = 0.456 (0.744)		χ²(2) = 0.36, p = 0.836 (0.919)	
Don’t know	121 (18.3)	304 (16.2)	14 (11.2)	27 (12.4)	6 (7.6)	12 (10.1)
Yes	309 (46.8)	946 (50.4)	81 (64.8)	151 (69.3)	50 (63.3)	73 (61.3)
No	230 (34.9)	629 (33.5)	30 (24)	40 (18.4)	23 (29.1)	34 (28.6)
Hearing aids	χ²(3)=1.01, p = 0.799 (0.827)		χ²(3)=1.04, p = 0.792 (0.88)		χ²(3)=1.2, p = 0.754 (0.919)	
Yes, on both ears	43 (6.5)	115 (6.1)	22 (17.6)	43 (19.7)	12 (15.2)	22 (18.5)
Yes, on the right ear	4 (0.6)	19 (1.0)	4 (3.2)	6 (2.8)	3 (3.8)	4 (3.4)
Yes, on the left ear	8 (1.2)	23 (1.2)	5 (4.0)	5 (2.3)	4 (5.1)	3 (2.5)
No	605 (91.7)	1722 (91.6)	94 (75.2)	164 (75.2)	60 (76.0)	90 (75.6)
**Problems tolerating sounds**	**χ²(4) = 70.08, p** < **0.0001 (0.0001)**		χ²(4) = 9.81, p = 0.044 (0.119)		χ²(3) = 5.15, p = 0.162 (0.485)	
Never	11 (1.7)	95 (5.1)	0 (0.0)	1 (0.5)	0 (0.0)	0 (0.0)
Rarely	61 (9.2)	347 (18.5)	4 (3.2)	16 (7.3)	2 (2.5)	9 (7.6)
Sometimes	248 (37.6)	742 (39.5)	24 (19.2)	66 (30.3)	14 (17.7)	31 (26.1)
Usually	207 (31.4)	469 (25)	49 (39.2)	73 (33.5)	28 (35.4)	39 (32.8)
Always	133 (20.2)	226 (12)	48 (38.4)	62 (28.4)	35 (44.3)	40 (33.6)
**Sounds cause pain or physical discomfort**	**χ²(2) = 51.96, p** < **0.0001 (0.0001)**		χ²(2) = 6, p = 0.05 (0.124)		χ²(2) = 0.69, p = 0.71 (0.919)	
Don’t know	39 (5.9)	115 (6.1)	7 (5.6)	16 (7.3)	3 (3.8)	7 (5.9)
Yes	427 (64.7)	919 (48.9)	98 (78.4)	144 (66.1)	62 (78.5)	88 (74)
No	194 (29.4)	845 (45)	20 (16)	58 (26.6)	14 (17.7)	24 (20.2)
**Temporomandibular problems**	**χ²(2) = 168.49, p** < **0.0001 (0.0001)**		**χ²(2) = 15.98, p** = **0 (0.002)**		**χ²(2) = 9.15, p** = **0.01 (0.077)**	
Don’t know	37 (5.6)	64 (3.4)	11 (8.8)	17 (7.8)	6 (7.6)	7 (5.9)
Yes	231 (35)	245 (13)	50 (40)	45 (20.6)	36 (45.6)	31 (26.1)
No	392 (59.4)	1570 (83.6)	64 (51.2)	156 (71.6)	37 (46.8)	81 (68.1)
**Vertigo/dizziness**	**χ²(2) = 177.98, p** < **0.0001 (0.0001)**		**χ²(2) = 38.25, p** < **0.0001 (0.0005)**		**χ²(2) = 24.39, p** < **0.0001 (0.002)**	
Don’t know	33 (5.0)	64 (3.4)	6 (4.8)	9 (4.1)	1 (1.3)	4 (3.4)
Yes	278 (42.1)	324 (17.2)	67 (53.6)	47 (21.6)	40 (50.6)	21 (17.7)
No	349 (52.9)	1491 (79.4)	52 (41.6)	162 (74.3)	38 (48.1)	94 (79.0)
**Neck pain**	**χ²(2) = 339.35, p** < **0.0001 (0.0001)**		**χ²(2) = 36.22, p** < **0.0001 (0.0005)**		**χ²(2) = 13.17, p** = **0.001 (0.014)**	
Don’t know	14 (2.1)	26 (1.4)	3 (2.4)	6 (2.8)	1 (1.3)	1 (0.8)
Yes	397 (60.2)	410 (21.8)	83 (66.4)	72 (33.0)	51 (64.6)	46 (38.7)
No	249 (37.7)	1443 (76.8)	39 (31.2)	140 (64.2)	27 (34.2)	72 (60.5)
**Other pain syndromes**	**χ²(2) = 145.17, p** < **0.0001 (0.0001)**		**χ²(2) = 33.37, p** < **0.0001 (0.0005)**		**χ²(2) = 17.49, p** = **0 (0.003)**	
Don’t know	24 (3.6)	18 (1.0)	4 (3.2)	2 (0.9)	3 (3.8)	3 (2.5)
Yes	256 (38.8)	341 (18.2)	71 (56.8)	60 (27.5)	43 (54.4)	31 (26.1)
No	380 (57.6)	1520 (80.9)	50 (40)	156 (71.6)	33 (41.8)	85 (71.4)
**Under psychatric treatment**	**χ²(2) = 34.43, p** < **0.0001 (0.0001)**		χ²(2) = 5.81, p = 0.055 (0.127)		χ²(1) = 3.94, p = 0.047 (0.204)	
Don’t know	7 (1.1)	9 (0.5)	0 (0.0)	1 (0.5)	0 (0.0)	0 (0.0)
Yes	89 (13.5)	122 (6.5)	28 (22.4)	28 (12.8)	21 (26.6)	18 (15.1)
No	564 (85.5)	1748 (93)	97 (77.6)	189 (86.7)	58 (73.4)	101 (84.9)
**Diagnosed disease**	**χ²(1) = 21.32, p** < **0.0001 (0.0001)**		**χ²(1) = 8.01, p** = **0.005 (0.023)**		χ²(1) = 2.15, p = 0.143 (0.485)	
Yes	265 (40.2)	570 (30.3)	60 (48)	71 (32.6)	34 (43)	39 (32.8)
No	395 (59.9)	1309 (69.7)	65 (52)	147 (67.4)	45 (57)	80 (67.2)

Pairwise comparisons using Pearson’s Chi-square test are reported. Percentages (%) displayed refer to column percentages. P values adjusted for multiple comparisons are shown between parenthesis. Estimates in bold are statistically significant at 0.05 level.

## Discussion

Our study shows a strong relationship between headache and tinnitus with increasing severity. The relationship we found for any tinnitus [OR = 2.19 (95% CI: 1.81–2.64)] is within the range of what has been reported for migraine in another cross-sectional study [multivariable-adjusted OR = 1.77 (1.36–2.30), n = 5729]^24^. The present work goes beyond this analysis by showing the increasing relationship between headache and tinnitus severity reaching an OR of 5.63 (4.10–7.72) in the group with tinnitus as a big problem, suggesting either that headaches contribute to the distress caused by tinnitus, or that tinnitus distress contribute to headaches – a directionality that remains to be investigated.

Our study goes in line with findings from Langguth *et al*.^18^ as we replicate a number of points: vertigo/dizziness, neck pain and other pain syndromes constitute a dominant part of the symptomatology of subjects experiencing headaches, when having tinnitus as a big problem, or in severe tinnitus. A significant number of individuals with vertigo and migraine headache with tinnitus may fulfil diagnostic criteria for migraine^41^ or vestibular migraine^42^ and the association between tinnitus and migraine deserves further studies. Our study however included in a large part subjects from the population, rather than patients recruited in a clinical context, as those from the Tinnitus Research Initiative. As a consequence, a greater number of variables were found to differ between tinnitus subjects with or without headaches as we included a larger sample of the population. The any tinnitus group indeed includes subjects experiencing tinnitus occasionally (47%) and permanently (53%), where differences in income and employment could be found, in perception, psychological features (stress, anxiety and depression), fear of tinnitus, hyperacusis and quality of life. These aspects could not all be captured in the study by Langguth and colleagues (2017) most likely due to the fact that as severity increases, fewer differences are seen between the headache and non-headache tinnitus groups. Here, the prevalence of headache in tinnitus subjects started at 26% for the any tinnitus group, up to 40% in the severe tinnitus group as defined by the THI scale. These findings are consistent with the notion that headache, and particularly migraine, could be a facilitator to the development of severe tinnitus, as suggested with the greater risk in developing clinically significant tinnitus with migraines [adjusted Hazard Ratio: 3.30 (2.17–5.00)]^26^ when compared to self-reported tinnitus of unknown severity [adjusted Recurrence Ratio: 1.28 (1.06, 1.56)]^43^.

In spite of the sample differences between STOP and the TRI database, the proportion of clinically significant tinnitus subjects with headache is higher in STOP (40%) than in the TRI (27%). Indeed, the county of Stockholm states in their regional guidelines a severe score based on the THI warrants a referral to specialist care^44^, which hence allows to infer that the severe tinnitus group presented here would be equivalent to clinically significant tinnitus. Differences between the two datasets could be either be regional or due to the fact that a broader range of troublesome tinnitus is included in the TRI. Unlike the Lannguth *et al*. study (2017), we were not able to perform the analysis according to the laterality of the headache, nor according to subtypes of headache (e.g. migraine, tension-type headache, cluster headache). There, painful sensations upon loud sound exposure were more frequent with bilateral headache, whereas cluster headache was the one most impacting on tinnitus distress. However, common to all types of headaches was the influence on comorbid vertigo, temporomandibular joint (TMJ) complaints, neck pain, and pain in general^18^, which are also observed in our study.

The frequent co-occurrence of pain symptoms in clinically significant tinnitus with comorbid TMJ complaints^16,17^ or headache^18^, is consistent with the hypothesis that tinnitus shares similar neurocircuitry to chronic pain, whereby frontostriatal gating would be affected^30^. Tinnitus could also emerge as a consequence of vestibular migraine, frequently observed in patients with episodic vertigo^45,46^. Additional mechanisms may occur such as an increased excitability of the trigeminal system^47^, also leading to TMJ and neck pain^16^. An increased occurrence of tinnitus in the family is observed with co-morbid headache, although this increase was not as large as what was found for TMJ complaints^17^. Recent evidence in twins and adoptees point towards a significant genetic contribution to tinnitus^48,49^. It is thus possible that the heritability of tinnitus shares common genetic mechanisms with that of pain and migraine. Indeed, genetics of migraine indicate a predisposition towards generalized neuronal excitability due to the inability to regulate glutamate availability^19^, as it has been recently suggested in a mouse model of tinnitus^50^. Recently, a genome-wide association studies (GWAS) of 375,000 individuals (including 59,674 cases) identified 38 susceptibility loci for migraine^51^. However, such large-scale studies in tinnitus are lacking and would be valuable to estimate the overlap between migraine and tinnitus^52,53^. However, while large biobanks with information on tinnitus do exist, the depth of the phenotyping is often limited which is why large biobanking efforts in ENT clinics have been encouraged^54,55^.

The phenotypic study on tinnitus-only subjects suggests a sex-bias, whereby the proportion of women with tinnitus increases from 44% to 66% in presence of headaches, as reported by Langguth *et al*. (2017). Similar sex-bias in women was also recently described in tinnitus with co-morbid TMJ complaints^16,17^. However, when non-tinnitus controls are included, the sex bias vanishes with instead a trend towards stronger relationship between tinnitus and headache in men, which emphasizes the need to include non-tinnitus controls to infer on sexual dimorphisms. The greater relationship in men did not significantly differ from that of women possibly due to the low sample size of the severe tinnitus groups, which warrants investigation in larger studies. Reasons for the increased proportion of women with tinnitus when reporting headache could also be due to the fact that migraine and TMJ disorders are more frequent in women (3:1 female to male ratio in migraine; and 2:1 in TMJ disorders)^56,57^. Headaches are indeed among the leading causes of years lived with disability in Scandinavian women^58^. It is interesting to note though that greater stress due to tinnitus has been reported in women^6,59^ and that a greater association of tinnitus with suicide attempts was found in women but not in men^7^, underlying that the greater psychological impact of tinnitus in women should not be disregarded. Our work encourages the need of including sex as a biological variable (SABV) in future tinnitus studies.

There are a number of limitations to our study, which include self-reported tinnitus and headaches. Both of them are complex and heterogeneous conditions that pre-empt clinical examination. Indeed, our study incorporates participants that may not be representative of the clinical setting. Self-reports may yield different prevalence estimates than diagnostic codes from medical records, the latter representing more a severe form of tinnitus for which medical care is sought. Reliance on self-report introduces the possibility of recall bias, that would be otherwise minimized when involving a physician. However, as regional guidelines recommend a referral to specialty care when a severe THI score is reached^44^, one could – at least in part – infer on the phenotypic characteristics that would be expected in the clinic. Secondly, while STOP participants were recruited from LifeGene, which is representative of the general population, it is unclear whether our findings can be generalized to that of the general population. Not all LifeGene participants with tinnitus joined STOP, and as a consequence, a recruitment bias may have occurred e.g. towards more participants seeking treatment options. Thirdly, as headaches and tinnitus were assessed at one single point in time, it is not possible to understand the direction of the observed relationship.

Overall, our study confirms that tinnitus with co-morbid headaches is accompanied with vertigo, neck and other pain syndromes. The association between headache and tinnitus increases as tinnitus becomes more severe. Thus, an interprofessional approach to care would be warranted in these cases. Future longitudinal studies investigating the conjunct impact of headache with TMJ disorders may offer additional insights into their additive or synergistic contribution to tinnitus.

## Materials and Methods

### Sample

Participants were invited to join the Swedish Tinnitus Outreach Project via social media channels and through partnerships with local cohorts, including LifeGene^60^. All participants above 18 years of age were eligible. Voluntary registration was done on a website from STOP (https://stop.ki.se). After providing informed consent for having their data stored in a database and analyzed, participants were invited to fill an online questionnaire, which was answered between November 2015 and January 2018. The first part of this study was performed on a subset of individuals with or without tinnitus who responded to the European School for Interdisciplinary Tinnitus Research – Screening Questionnaire (ESIT-SQ) to investigate the association between headaches and tinnitus. The second part of this study focused only on tinnitus subjects with or without headaches in order to investigate their phenotypic traits. We followed the STROBE guidelines and the checklist is available in the online materials. All subjects gave written informed consent in accordance with the Declaration of Helsinki. The project has been approved by the Regional Ethics Review Board in Stockholm (2015/2129-31/1).

### Association study

The ESIT-SQ was designed to be comprehensive with a specific attention to risk factors that could be answered by people with or without tinnitus^61^. Here the tinnitus question A17 was: *“Tinnitus refers to the perception of noise in your head or ears (such as ringing or buzzing) in the absence of any corresponding source of sound external to your head. Over the past year, have you had tinnitus in your head or in one or both ears that lasts for more than five minutes at a time?”* with answer options being: “do not know”, “no never”, “no, not in the past year”, “yes, some of the time”, “yes a lot of time”, “yes most of the time”. Those answering “don’t know” or “no, not in the past year” were excluded from the analysis. Question A15 stated “*Do you suffer from any of the following pain syndromes?*” of which *Headache* was one of the options. For the present analysis, we thus included 1984 tinnitus cases and 1661 subjects without tinnitus from the STOP project.

### Tinnitus phenotyping study

The online survey consisted of a combination of standardized questionnaires translated in Swedish^40^. Participants were asked the question: “*Do you have tinnitus?*” with possible answers being: “do not know”, “no”, “yes, occasionally (now and then)”, “yes, always (all the time)”. All of those answering “yes, occasionally” or “yes, always” were considered in this analysis and represent the “any tinnitus” group. The Tinnitus Sample Case History Questionnaire (TSCHQ) measures phenotypic characteristics that may be associated with tinnitus^62^, and question #30 “*Do you suffer from headache?*” was adapted in Swedish leading to an intra-class coherent coefficient of 0.77 (good) in a test-retest^40^. The Tinnitus Handicap Inventory (THI)^63^, the Tinnitus Functional Index (TFI)^64^, the Fear of Tinnitus Questionnaire (FTQ)^65^, the Tinnitus Catastrophizing Scale (TCS)^65^, the Hyperacusis Questionnaire (HQ)^66^, the Perceived Stress Questionnaire (PSQ)^67^, the Hospital Anxiety Depression Scales for Anxiety (HADS A) and depression (HADS D)^68^, and the World Health Organization’s Quality of Life (WHOQoL)-BREF^69^ were used. Adaptation of these questionnaires to Swedish and their validity has been reported elsewehere^40^. Numerical Rating Scales (NRS) for Loudness, Awareness, and Annoyance were obtained via the TSCHQ (questions 12, 16, and 17). Severe tinnitus was operationally defined in two ways. The first used a revised grading system of the original eight-factor 25 item of the TFI^70^ where a TFI cut-off score ≥ 48 denotes a big problem. The second used a THI cut-off score ≥ 58, since this boundary is used as a criterion for referral to specialty care in the Stockholm County^44^, here referred to as severe tinnitus according to the terminology used in the THI. Importantly, these two groups both contain the most severe tinnitus cases according to two commonly used clinical tools. Overlap between the groups is expected. We present both groups to maximize comparability with other studies and minimize selection bias that could be introduced from choosing one tool for evaluation. For the present analysis we included 1,879 subjects with tinnitus without headache and 660 subjects with tinnitus with headache.

### Statistical analysis

The statistical approach used in the tinnitus phenotyping study has been described elsewhere^17^. Briefly, phenotypic characteristics such as tinnitus loudness, pitch, onset, whether tinnitus is pulsating or not, what the tinnitus sounds like, and onset-related events were obtained from the TSCHQ. Additional sociodemographic data (i.e. marital status, income, employment status, and education level) were obtained using questions from Svensson *et al*.^71^. All statistical analyses were performed in JMP 13 (SAS Institute Inc.) and R (R Core Team, 2019). For nominal variables, Pearson’s Chi-squared test was used. Homoscedasticity between groups was tested for using the Brown-Forsythe test and showed significant differences between subgroups for multiple variables. Multiple questionnaire total scores also deviated from a normal distribution. The non-parametric Wilcoxon’s test and Cliffs delta (δ) for effect size, using the ‘effsize’ package in R, were used for all comparisons to provide easy comparability between different groups for the reader. In order to investigate the potential impact of multiple comparisons on the discovery rates of our tests, we also report p-values adjusted by the method of Benjamini and Hochberg. The adjustments were computed for each set of p-values resulting from multiple tests carried out.

For the association study, we estimated the odds ratios (ORs) and corresponding 95% confidence intervals (CIs) for tinnitus using unconditional multiple logistic regression models after adjustment for sex, age, educational level (model 1) and also self-reported hearing ability (ESIT-SQ question A13) (model 2). Logistic regressions were performed in SAS 9.4 (SAS Institute, Cary, North Carolina).

## Supplementary information

Supplementary information.

## Data Availability

The datasets generated during and/or analysed during the current study are available from the corresponding author on reasonable request.
